# An H5N1 M2e-based multiple antigenic peptide vaccine confers heterosubtypic protection from lethal infection with pandemic 2009 H1N1 virus

**DOI:** 10.1186/1743-422X-7-151

**Published:** 2010-07-12

**Authors:** Guangyu Zhao, Shihui Sun, Lanying Du, Wenjun Xiao, Zhitao Ru, Zhihua Kou, Yan Guo, Hong Yu, Shibo Jiang, Yuchun Lone, Bo-Jian Zheng, Yusen Zhou

**Affiliations:** 1 State Key Laboratory of Pathogen and Biosecurity, Beijing Institute of Microbiology and Epidemiology, Beijing 100071, China; 2 Lindsley F. Kimball Research Institute, New York Blood Center, New York, NY 10065, USA; 3 U.1014 (Ex 542), INSERM, Université Paris-Sud, Hôpital Paul Brousse, Villejuif 94800, France; 4 Department of Microbiology, The University of Hong Kong, Hong Kong, China

## Abstract

**Background:**

A 2009 global influenza pandemic caused by a novel swine-origin H1N1 influenza A virus has posted an increasing threat of a potential pandemic by the highly pathogenic avian influenza (HPAI) H5N1 virus, driving us to develop an influenza vaccine which confers cross-protection against both H5N1 and H1N1 viruses. Previously, we have shown that a tetra-branched multiple antigenic peptide (MAP) vaccine based on the extracellular domain of M2 protein (M2e) from H5N1 virus (H5N1-M2e-MAP) induced strong immune responses and cross-protection against different clades of HPAI H5N1 viruses. In this report, we investigated whether such M2e-MAP presenting the H5N1-M2e consensus sequence can afford heterosubtypic protection from lethal challenge with the pandemic 2009 H1N1 virus.

**Results:**

Our results demonstrated that H5N1-M2e-MAP plus Freund's or aluminum adjuvant induced strong cross-reactive IgG antibody responses against M2e of the pandemic H1N1 virus which contains one amino acid variation with M2e of H5N1 at position 13. These cross-reactive antibodies may maintain for 6 months and bounced back quickly to the previous high level after the 2nd boost administered 2 weeks before virus challenge. H5N1-M2e-MAP could afford heterosubtypic protection against lethal challenge with pandemic H1N1 virus, showing significant decrease of viral replications and obvious alleviation of histopathological damages in the challenged mouse lungs. 100% and 80% of the H5N1-M2e-MAP-vaccinated mice with Freund's and aluminum adjuvant, respectively, survived the lethal challenge with pandemic H1N1 virus.

**Conclusions:**

Our results suggest that H5N1-M2e-MAP has a great potential to prevent the threat from re-emergence of pandemic H1N1 influenza and possible novel influenza pandemic due to the reassortment of HPAI H5N1 virus with the 2009 swine-origin H1N1 influenza virus.

## Background

At the same time concern was raised about a possible pandemic resulting from the highly pathogenic avian influenza (HPAI) H5N1 virus, the world confronted the first influenza pandemic of the 21st century caused by a novel influenza A H1N1 virus [1]. This pandemic H1N1 virus was first identified in April 2009 and demonstrated a rapid rate of spread. As of 9 May, 2010, WHO has reported at least 18,036 fatal cases in more than 214 countries [2]. Although H5N1 virus has not yet evolved to become transmissible among humans, it has still presented a high mortality rate of approximately 60% [3]. Consequently, WHO considers the H5N1 virus to be a potential human pandemic [4]. Antigenic and genetic analysis has suggested that the current pandemic 2009 H1N1 virus is a product of reassortment between genes in the human, avian and swine influenza strains [5]. There is a concern that one more human pandemic influenza virus could be derived in the future from animal reservoirs, such as avian, possessing both rapid interpersonal transmissibility and high lethality. The potential shortage of pandemic influenza vaccines and the absence of specific-immunity in the human population make the development of a cross-protective influenza vaccine, which is based on conserved antigens, a promising prophylactic strategy.

The extracellular domain of influenza M2 protein (M2e) is highly conserved across influenza A subtypes and has become an attractive antigen target for producing a cross-protective influenza vaccine with broad-spectrum prevention [6]. To date, many groups have reported M2e-based vaccine candidates in different forms such as virus-like particles [7-9], recombinant proteins [10-12], DNA [13], and synthetic peptides [14,15]. However, most of these M2e vaccines require either chemical or genetic strategies to form M2e-carrier fusion constructs in order to overcome the poor immunogenicity of M2e alone. Therefore, multiple antigenic peptides (MAPs) would provide an ideal platform for the application of a vaccine based on a short peptide antigen, such as M2e. This type of construct would result in a large macromolecule with a high molar ratio of target peptide antigen to a small immunologically inert core molecule without requiring further conjugation to a carrier protein [16,17]. In our previous study, we designed and synthesized a MAP with radially branching lysine dendrites onto which four copies of conserved M2e of H5N1 virus were attached. Such H5N1-M2e-MAP vaccine elicited high titers of H5N1-M2e-specific serum antibodies and conferred efficacious protection against different clades of H5N1 virus [18]. Here, we further proved that the H5N1-M2e-MAP candidate vaccine can provide heterosubtypic protection against lethal infection of pandemic 2009 H1N1 virus.

## Materials and methods

### Mice

Six- to eight-week-old female BALB/c mice were purchased from the Beijing Animal Center (Beijing, China). All mice were maintained in a specific pathogen-free facility and housed in cages containing sterilized feed, autoclaved bedding and water. The animal study was approved by the Institutional Animal Care and Use Committee (IACUC).

### Virus

The pandemic 2009 H1N1 virus used in this study is A/Beijing/501/09 isolated from a confirmed H1N1 case in China. Virus was grown in the allantoic cavities of 10-day-old embryonated chicken eggs. Virus-containing allantoic fluid was harvested and stored in aliquots at -80°C until use. The 50% lethal dose (LD_50_) was determined in mice after serial dilutions of the virus stock. All infectious experiments were performed in an approved biosafety level 3 (BSL-3) facility.

### H5N1-M2e-MAP

**Figure 1:**
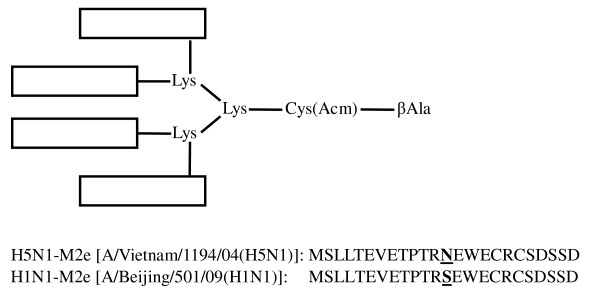
**Structure of the synthetic H5N1-M2e-MAP**. The M2e-MAP was synthesized on [Fmoc-Lys(Fmoc)]_2_-Lys-Cys(Acm)-βAla-Wang Resin in a tetra-branched form, which carries four copies of H5N1-M2e. The amino acid sequences of H5N1-M2e derived from A/Vietnam/1194/04(H5N1) and the H1N1-M2e sequence from A/Beijing/501/09(H1N1) are respectively listed at the bottom of this figure. The difference between H1N1-M2e and H5N1-M2e is bolded and underlined.

As described previously [18], tetra-branched multiple antigenic peptide carrying four copies of M2e peptide of H5N1 virus (H5N1-M2e-MAP) was synthesized on [Fmoc-Lys(Fmoc)]_2_-Lys-Cys(Acm)-βAla-Wang Resin (Advanced ChemTech, Louisville, Kentucky, USA) on a 0.02 mM scale using an Applied Biosystems model 433A peptide synthesizer. Cleavage of the peptide from the resin was performed by treatment with trifluoroacetic acid (TFA), DTT, water, and triisopropylsilane (TIPS) in the ratio 88:5:5:2 (TFA/DTT/H_2_O/TIPS). Crude peptide was purified by reversed phase high-performance liquid chromatography (RP-HPLC). The structure of H5N1-M2e-MAP is shown in Fig. 1.

### Immunization and virus challenge

Mice were vaccinated subcutaneously (s.c.) with 10 μg of H5N1-M2e-MAP in the presence of Freund's complete adjuvant (Sigma, MO) or intramuscularly (i.m.) plus aluminum adjuvant (Sigma). The 1^st ^boost was given with the same amount of H5N1-M2e-MAP in Freund's incomplete adjuvant (Sigma) or aluminum adjuvant three weeks later. The mice received the 2^nd ^boost six months later. Mice injected with Freund's or aluminum adjuvant alone were used as the respective control. Sera were collected at 1 week and 3 and 6 months after 1^st ^boost and 1 week after 2^nd ^boost to detect cross-reactive antibodies against H1N1-M2e. Sera collected before immunization were used as negative controls. Two weeks after 2^nd ^boost, mice were intraperitoneally (i.p.) anesthetized with ketamine (75 mg/kg) and intranasally (i.n.) challenged with a lethal dose (10LD_50_) of A/Beijing/501/09. Infected mice were observed and weighed daily for 2 weeks. Lung tissues were collected from euthanized mice 3 days post-challenge for further virological testing and histopathological analysis.

### ELISA

The cross-reactivity of H5N1-M2e-MAP-induced antibody against H1N1-M2e was detected by ELISA. Briefly, 96-well microtiter plates were pre-coated with 10 μg/ml of H1N1-M2e peptide (SBS Genetech, Beijing, China) overnight at 4°C. After blocking with 5% BSA containing 0.05% Tween-20 in PBS, serial diluted mouse sera were added to the plates, followed by addition of HRP-conjugated rabbit anti-mouse IgG (1:2,000, Invitrogen, Carlsbad, CA) at 37°C for 0.5 h. Assay was developed using 3,3',5,5'-tetramethylbenzidine (TMB) (Zymed, Carlsbad, CA), and the reaction was stopped by adding 1N H_2_SO_4_. The absorbance at 450 nm was measured by an ELISA plate reader (Sunrise™ microplate reader, TECAN, NC).

### Viral titers in lung tissues

Viral titers in lungs were determined by 50% Tissue culture infective dose **(**TCID_50_) as described [18], except that TPCK-trypsin (Sigma) was added into the medium (2 μg/ml), and 0.5% chicken erythrocytes were used in hemagglutination confirming cytopathic effect (CPE) endpoint.

### Histopathological analysis

The lung tissues of challenged mice were immediately fixed in 10% neutral buffered formalin and embedded in paraffin wax. Sections were made at 4 - 6 μm thickness and mounted on slides. Histopathological changes were examined by H & E staining and observed under light microscopy.

### Statistical analysis

The significance between survival curves was analyzed by Kaplan-Meier survival analysis with log-rank test. Other data were analyzed using the 2-tailed Student's t test. *P *< 0.001 was considered significant. All analyses were performed in Graphpad Prism software.

## Results

### H5N1-M2e-MAP vaccination induced high titers of cross-reactive antibody against H1N1-M2e peptide

**Figure 2:**
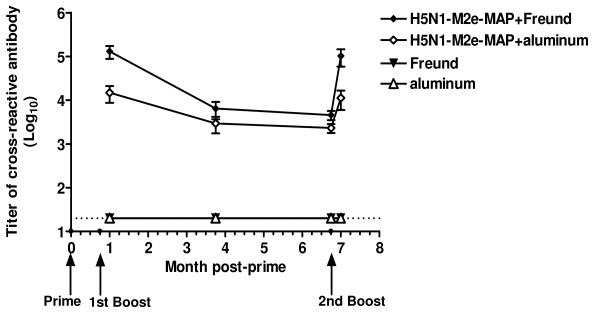
**Cross-reactivity of H5N1-M2e-MAP-induced antibody against H1N1-M2e peptide**. Mice were primed and boosted with H5N1-M2e-MAP vaccine and sera were collected as described in Materials and Methods to detect cross-reactivity against H1N1-M2e by ELISA. The end-point titer of each sample was determined as the highest dilution that yielded an OD_450 nm _value greater than twice of that from pre-vaccination. The data are expressed as mean ± standard deviation (SD) of 10 mice per group. The lower limit of detection (1:20) is indicated by a dotted line. Time points of immunizations are shown as small spots on X-axis, and indicated by arrows at the bottom.

The M2e of human influenza virus is significantly different from that of avian influenza virus [19]. However, the pandemic 2009 H1N1 virus and H5N1 virus share a similar M2e sequence, except for one amino acid, as indicated in Fig. 1 with representative virus stains. The serological examination illustrated the cross-reactivity of H5N1-M2e-MAP-induced antibodies that recognized H1N1-M2e. As shown in Fig. 2, one week after the 1^st ^boost, H5N1-M2e-MAP in Freund's adjuvant or aluminum adjuvant elicited strong cross-reactive IgG antibody against H1N1-M2e with the titer reaching 1:10^5 ^or 1:10^4^, respectively. Despite a slight decrease in the following six months, the titers of cross-reactive antibody bounced back quickly to the previous high level after the 2^nd ^boost. In contrast, only background level of anti-H1N1-M2e antibody response was detected in Freund's or aluminum adjuvant controls. These results suggest that H5N1-M2e-MAP can induce long-term and potent antibody responses that may be effective against both homologous HPAI H5N1 virus and heterologous pandemic 2009 H1N1 virus.

### H5N1-M2e-MAP vaccination limited replication of pandemic 2009 H1N1 and attenuated virus-induced lung pathology

**Figure 3:**
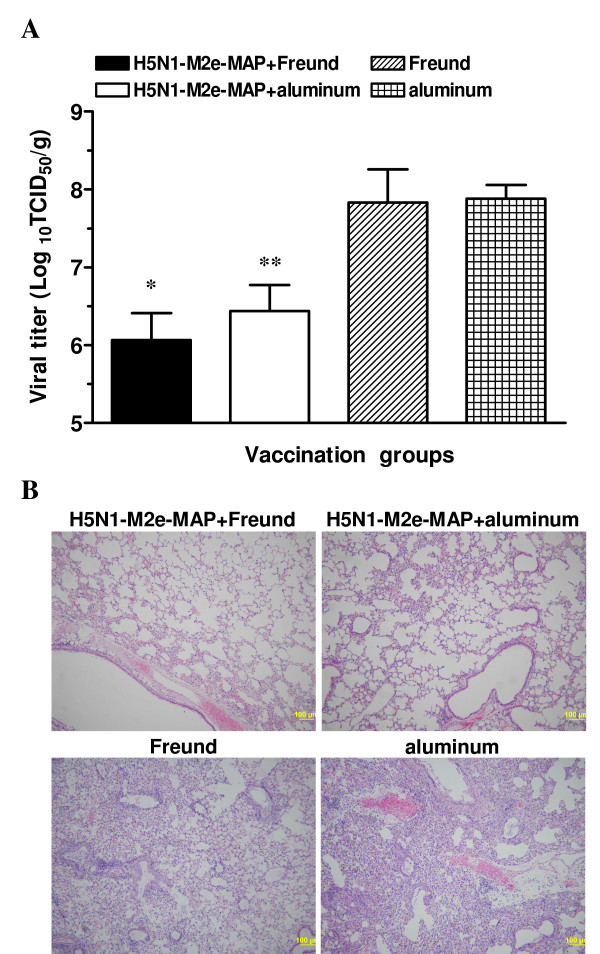
**Detection of viral titers and histopathological changes in lungs of the H5N1-M2e-MAP-vaccinated mice following heterosubtypic challenge with pandemic 2009 H1N1 virus**. Two weeks post-last boost, H5N1-M2e-MAP-vaccinated mice were challenged with lethal dose (10LD_50_) of pandemic 2009 H1N1 virus A/Beijing/501/09 strain, and lung tissues were collected three days later. (A) Viral titers in lungs of infected mice. The data are expressed as Log_10_TCID_50_/g of lung tissues, and presented as GMT ± SD of 5 mice per group. * indicates *P *< 0.001 compared to the Freund's adjuvant control; ** means *P *< 0.001 compared to the aluminum adjuvant control. Experiments were repeated three times. (B) Histopathological changes in the lungs following virus challenge. The figure indicates the representative images of histopathological damage from H & E-stained lungs of 5 mice per group (magnification, 100×).

In our established mouse model, the virus replicated in the lung tissue to the peak, reaching the highest titer three days post-lethal infection of pandemic 2009 H1N1 virus (data not shown). To determine whether the H5N1-M2e-MAP-induced immune responses confer heterosubtypic protection against infection with pandemic 2009 H1N1 virus, the vaccinated mice were sacrificed three days after lethal virus challenge (10LD_50_) of A/Beijing/501/09 and lung tissues were collected for detection of viral titers and histopathological examination. Compared with the matched adjuvant control group, the average viral titer in lungs of H5N1-M2e-MAP-vaccinated mice was significantly lower (*P *< 0.001) after lethal challenge with the pandemic 2009 H1N1 virus (Fig. 3A), suggesting that the H5N1-M2e-MAP can induce potent protective immunity against viral replication following pandemic 2009 H1N1 virus infection.

Further examination of the lung tissues of virus-challenged mice revealed that dramatic lung damages mainly occurred in the control mice, which were characterized by diffused alveolar lesion with pneumocytes denatured, alveolar macrophages and cellular debris mixed with fibrin in alveolar lumina, and widened lung septa with the infiltration of lymphocytes and a few neutrophils. The bronchiolar epithelium was degenerated and collapsed with multifocal peribronchiolar infiltration of lymphocytes and a few neutrophils. The blood vessel endothelium was damaged with moderate edema and infiltration of lymphocytes and neutrophils in the periendothelium. However, lungs of H5N1-M2e-MAP-vaccinated mice exhibited fewer histopathological changes, with only mild pulmonary interstitial pneumonia and moderate lymphocytic infiltration (Fig. 3B). The above data implied that H5N1-M2e-MAP vaccination protects mice against lethal infection with the pandemic 2009 H1N1 virus through a combination of limiting viral replication in the lungs and attenuating virus-induced lung pathology.

### H5N1-M2e-MAP vaccination provided effective heterosubtypic protection from lethal challenge with pandemic 2009 H1N1 virus

**Figure 4:**
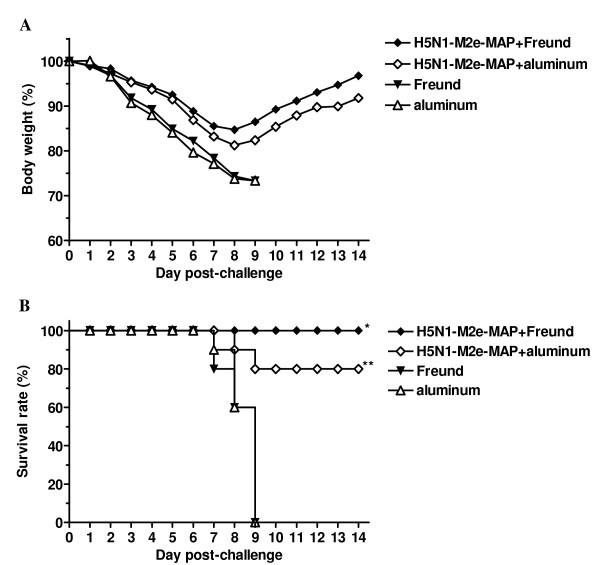
**Cross-protection of H5N1-M2e-MAP-vaccinated mice against lethal challenge of pandemic 2009 H1N1 virus**. H5N1-M2e-MAP-vaccinated mice were challenged with lethal dose (10LD_50_) of heterosubtypic H1N1 virus A/Beijing/501/09 strain and monitored daily for 2 weeks post-challenge. (A) Percentage change (%) of mouse body weight. Each point represents mean body weight of 10 mice per group. (B) Survival rate (%). The significant differences (*P *< 0.001) of H5N1-M2e-MAP plus Freund's adjuvant versus Freund's adjuvant is indicated as *, while H5N1-M2e-MAP plus aluminum adjuvant versus aluminum adjuvant is indicated as **.

To further confirm cross-protection conferred by H5N1-M2e-MAP against heterologous infection with the pandemic 2009 H1N1 virus, mice received lethal challenge (10LD_50_) of A/Beijing/501/09 strain were monitored for weight loss and death for the subsequent two weeks post-challenge. As shown in Fig. 4A, mice vaccinated with H5N1-M2e-MAP in Freund's or aluminum adjuvant rallied from body weight loss eight days after virus infection. In contrast, body weight of the mice in the adjuvant control group dramatically decreased, even more than 25%, in some cases. All mice received adjuvant alone died within 10 days after lethal virus challenge. In comparison, 100% and 80% of the mice survived in the groups vaccinated with H5N1-M2e-MAP in Freund's and aluminum adjuvants, respectively, with the survival rate significantly different from the matched adjuvant control (*P *< 0.001) (Fig. 4B). These data illustrated that H5N1-M2e-MAP can afford heterosubtypic protection against lethal challenge of pandemic 2009 H1N1 virus.

## Discussion

To develop safe and effective prophylactic strategies to combat human infections by both pandemic 2009 H1N1 virus and H5N1 virus, the highly conserved M2e of influenza A virus has proven to be a promising target antigen to produce cross-protective influenza vaccines. In this study, H5N1-M2e-MAP, whose cross-protection against different clades of H5N1 virus has already been established in our previous report [18], was shown to confer heterosubtypic protection from lethal infection with the pandemic 2009 H1N1 virus. Remarkably, all mice vaccinated with H5N1-M2e-MAP plus Freund's adjuvant survived the lethal heterologous virus challenge. Although there is partial protection (80%) in the H5N1-M2e-MAP plus aluminum adjuvant group, H5N1-M2e-MAP vaccination in both Freund's and aluminum adjuvants demonstrated similar efficacy in limiting viral replication and attenuating virus-producing histopathological damage in lung tissues, thus showing the ability to control disease transmission.

Notably, it seems that higher titers of cross-reactive antibodies against H1N1-M2e result in better heterosubtypic protection from lethal virus infection (Figs. 2 and 4), implying that M2e-induced cross-reactive antibody is a crucial component in heterosubtypic protection in M2e-based vaccines. Other groups have reported that the antiviral effect of M2e-based vaccines was mediated by antibodies to M2e antigen and that its mechanism was antibody-dependent cell-mediated cytotoxicity (ADCC) and/or complement-mediated cytotoxicity (CDC) [20,21]. Therefore, the cross-protection of the M2e vaccine was based on the premise that antibody responses with high levels of cross-reactivity were induced following vaccination. However, the difference of M2e amino acid sequences between human-type and avian-type viruses would affect their mutual recognition in varying degrees. Specifically, Fan et al. [14] reported that antisera against human-type M2e sequence failed to react with avian-type M2e peptide. Also, Liu et al. [22] indicated that mAb specific to a region (aa 6 - 13) of human-type M2e sequence can only weakly recognize, or not recognize at all, avian-type M2e sequence with variations in the same range. Nevertheless, in our studies, H5N1-M2e-MAP not only induced high titers of specific antibody against H5N1-M2e [18] but also elicited potent and prolonged cross-reactive antibody recognizing H1N1-M2e (Fig. 2). Although it is not certain whether the only amino acid difference between the H1N1-M2e and H5N1-M2e (as indicated in Fig. 1) is located outside the region containing B-cell epitopes, the induction of cross-reactive antibody by H5N1-M2e-MAP against H1N1-M2e is obvious, and it is confirmed that H5N1-M2e-MAP can afford heterosubtypic protection against pandemic 2009 H1N1 virus (Figs. 3 and 4), regardless of the difference in M2e sequence between H1N1 and H5Nl by one amino acid.

Given that the immunization regimen consisting of H5N1-M2e-MAP and aluminum, the only adjuvant approved for use in humans, induced weaker immune responses (Fig. 2) and protective immunity (Fig. 4) than that is comprised of H5N1-M2e-MAP and Freund's adjuvant, it is urgently needed to develop more effective and safe adjuvant than aluminum for clinical use of human vaccines.

## Conclusions

The highly conserved M2e is an appropriate antigen target for the development of a cross-protective influenza vaccine. The present study revealed that the H5N1-M2e-MAP vaccine, whose cross-clade protection against divergent H5N1 viruses was confirmed in our previous report, can afford heterosubtypic protection against pandemic 2009 H1N1 virus, supporting the concept of an M2e-based vaccine with broad-spectrum protection against both the existing influenza virus and the emergent variant.

## Competing interests

The authors declare that they have no competing interests.

## Authors' contributions

GZ and YZ designed research. GZ, SS, WX, YG, and HY performed research. GZ, SS, ZR and ZK analyzed data. GZ, SS, LD, SJ, YL, BZ and YZ wrote and modified the paper. GZ and SS have equal contributions to this paper. All authors read and approved the final manuscript.
